# Association between dietary inflammatory index and cognitive impairment among American elderly: a cross-sectional study

**DOI:** 10.3389/fnagi.2024.1371873

**Published:** 2024-03-14

**Authors:** Yu Zhang, Yuanyuan Peng, Wei Deng, Qian Xiang, Wang Zhang, Maohang Liu

**Affiliations:** ^1^Chongqing Mental Health Center, Chongqing, China; ^2^Department of Critical Care Medicine, Chongqing General Hospital, Chongqing University, Chongqing, China

**Keywords:** dietary inflammatory index, cognitive impairment, cognitive performance, inflammation, elderly, National Health and Nutrition Examination Survey

## Abstract

**Background:**

It has been shown that inflammation may be associated with cognitive impairment (CI). Diet modulates inflammation. However, there is currently a scarcity of epidemiological studies exploring the connection between the inflammatory potential of diet and CI. The objective was to investigate the correlation between the dietary inflammatory index (DII) and cognitive impairment in older adults in the United States.

**Methods:**

The present investigation utilized a cross-sectional dataset obtained from the National Health and Nutrition Examination Survey (NHANES) from 2011 to 2014. Dietary intake data was used to calculate DII scores, which were then used to categorize participants into quartiles. Participants’ cognitive function was assessed using the Consortium to Establish a Registry for Alzheimer’s Disease (CERAD), Animal Fluency Test (AFT), and Digit Symbol Substitution Test (DSST). Individuals who scored in the lowest quartile on any of these tests were classified as exhibiting low cognitive performance. The association between DII and cognitive impairment was investigated by multivariate logistic regression, smooth curve fitting, and subgroup analysis.

**Results:**

A total of 947 older adults were enrolled in the study. Following the adjustment of confounding variables, DII scores exhibited a significant and positive correlation with low cognitive performance, as measured by AFT (OR 1.15, 95% CI 1.02–1.28, *p =* 0.02) and the DSST (OR 1.38, 95% CI 1.13–1.68, *p =* 0.004). Compared with the lowest quartile of DII, the highest weighted odds ratio of cognitive impairment based on AFT was observed in the fourth quartile group (OR 1.89, 95% CI 1.05–3.38, *p =* 0.03). Similarly, a comparable pattern was evident in DSST (OR 4.30, 95% CI 1.85–9.98, *p =* 0.003). Additionally, the smooth curve fitting results showed a nonlinear relationship between DII and cognitive decline evaluated by DSST (*p* for nonlinearity = 0.016). No interaction effects between cognitive impairment and age or gender were observed in relation to all cognitive test scores.

**Conclusion:**

This research reveals a positive link between diet with higher inflammatory potential and cognitive decline among elderly individuals in America. However, additional studies on dietary interventions are necessary to explore the cause-and-effect relationship.

## 1 Introduction

Cognitive impairment (CI) refers to the loss or reduction of cognitive functions, including memory, language, attention, problem-solving, and executive function. CI encompasses a range of conditions, including mild cognitive impairment (MCI) and various other forms of dementia spanning a spectrum from mild to severe. With the population aging, the global prevalence of CI has emerged as a significant health concern faced by the aging demographic on a worldwide scale. In America, it is projected that the patient count afflicted with MCI and Alzheimer’s disease will rise from an estimated 18.3 million in 2020 to 35.4 million by 2060 (Rajan et al., 2021). CI often results in difficulties in daily functioning, and a higher likelihood of developing other health complications, resulting in an inability for individuals to live independently over an extended time. A lack of effective therapeutic agents has exacerbated the expected increase in healthcare and caregiving costs, which imposes a substantial financial burden (Vaz and Silvestre, 2020). Hence, mitigating risk factors linked to cognitive impairment is imperative to forestall its development.

One potential biological correlate to CI is systemic immune abnormalities. In the general population, chronic, low-grade inflammation and immune activation pose a risk for CI (Wang et al., 2022; Leonardo and Fregni, 2023). Diet is considered as an essential approach to managing the immune system, several nutrients and dietary patterns have shown the potential to modulate the inflammatory status. For instance, increased consumption of red meat and fried dishes, as well as reduced intake of whole grains, have been associated with an elevated prevalence of pro-inflammatory indicators in plasma (Tayyem et al., 2021). Whereas, polyphenols and antioxidant vitamins were identified as associated with decreased oxidative stress and neuroinflammation (Monacelli et al., 2017). Therefore, dietary patterns may be a valuable tool to mitigate the related risk of CI. The dietary inflammatory index (DII) has been extensively utilized in evaluating the inflammatory capacity of dietary patterns while investigating associations between diet-induced inflammation and various diseases. There is consistent evidence from animal studies regarding the impact of various dietary patterns on cognitive function (Morello et al., 2018; Liu et al., 2021; Zhuang et al., 2022). Nevertheless, there is a lack of epidemiological studies investigating the association between DII and CI. Therefore, we aimed to investigate the association between DII and CI in the American elderly population, which may provide evidence for exploring dietary preventive measures for cognitive impairments.

## 2 Materials and methods

### 2.1 Study population

The study population is derived from the National Health and Nutrition Examination Survey (NHANES), a cross-sectional study conducted biennially by the Centers for Disease Control and Prevention (CDC) in the United States. The survey’s methodology and materials underwent ethical scrutiny and received approval from the Ethics Review Board of the National Center for Health Statistics. Prior to both the interviews and examinations, all participants provided informed consent. Thus, this study did not require Institutional Review Board approval.

**Figure 1:**
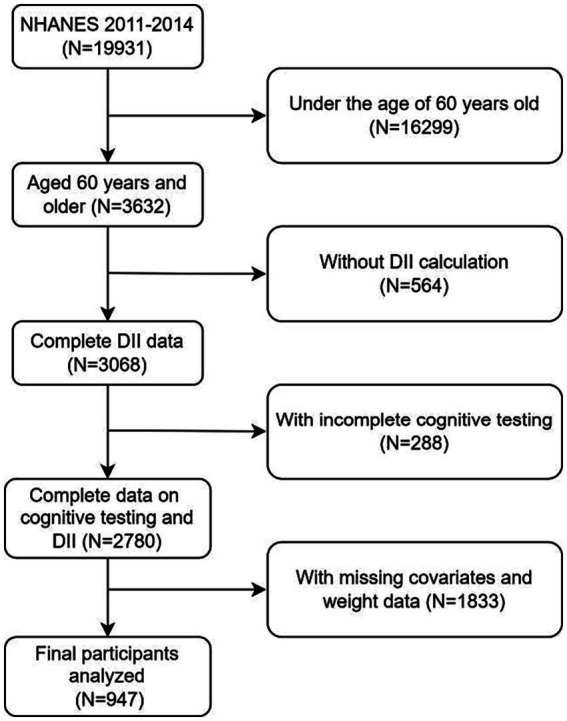
Flowchart for the selection of eligible participants.

Emerged data from NHANES 2011–2014 cycles were analyzed, including an aggregate of 19,931 participants initially. The exclusion criteria for this study encompassed the following: (a) individuals aged below 60 years old (*n* = 16,299), (b) individuals lacking dietary data required for DII calculation (*n* = 564), (c) individuals with incomplete cognitive testing (*n* = 288), (d) individuals with missing covariates and weight data (*n* = 1,833). In total, 947 individuals qualified for the analysis, as shown in Figure 1.

### 2.2 Calculation of DII

The dietary inflammation index (DII) was used as a special parameter in this study to evaluate overall dietary inflammation. The calculation of DII followed the scoring method established and improved by Shivappa et al. (2014). To calculate the DII, a 24-h dietary recall interview was conducted, capturing the consumption of 28 components for each participant. These components included energy, carbohydrate, protein, fiber, cholesterol, β-carotene, fatty acid, saturated fatty acid, monounsaturated fatty acids (MUFA), polyunsaturated fatty acids (PUFA), n3 polyunsaturated fatty acid, n6 polyunsaturated fatty acid, niacin, folic acid, magnesium, iron, zinc, selenium, caffeine, alcohol, and vitamins A, B1, B2, B6, B12, C, D, and E. To calculate the individual’s DII score, the standard mean of each food parameter was subtracted from the participant’s intake value, and then divided by the standard deviation. The individual’s Z-score value was measured and converted to centered proportions. These proportions were then multiplied by the corresponding inflammatory effect index and summed up, resulting in a total DII score for each participant. A higher positive DII score indicates a diet that promotes inflammation, while a lower value indicates a diet that has anti-inflammatory effects.

### 2.3 Cognitive performance assessment

The evaluation of cognitive performance was conducted on participants aged over 60 years using the following three testing methods: the CERAD test evaluates the immediate and delayed acquisition of novel language information. It is comprised of three successive learning trials and a delayed-recall trial with a cumulative score ranging from 0 to 40 (Morris et al., 1989). The AFT was employed to investigate verbal fluency within specific categories. Points are given based on the number of animals remembered within a 1-min interval (Carone, 2007). The DSST gauges processing speed, sustained attention, and working memory (Ryan and Lopez, 2001). In this evaluation, participants utilize a paper chart containing nine pairs of number symbols and are given 2 min to transcribe the symbols into 133 adjacent boxes alongside the numbers. The score, which can range from 0 to 133, reflects the cumulative count of correct matches. Higher scores correspond to enhanced cognitive performance across all assessments.

At present, there is no universally accepted criterion to determine cognitive impairment in relation to the CERAD, AFT, and DSST. As a result, we opted to employ the lowest quartile of each test score as the threshold to detect instances of cognitive impairment. This approach remains consistent with methodologies employed in prior published literature (Chen et al., 2017; Dong et al., 2020; Kou et al., 2023).

### 2.4 Covariates

Some potential confounding factors were extracted, including age, gender, race, education, body mass index (BMI), alcohol use, smoke. Poverty income ratio (PIR), with values categorized as either <1 or ≥ 1. This study also extracted information on comorbidities, including self-reported stroke. Hypertension is defined as a blood pressure of 140/90 mmHg or higher, a clinical diagnosis of hypertension, or the use of prescribed anti-hypertensive medication as reported in the health questionnaire. Diabetes was diagnosed if participants fulfilled at least one of the following criteria: (a) a clinical diagnosis of diabetes confirmed by medical professional, (b) the administration of medication or insulin for diabetes management, (c) a glycohemoglobin A1c (HbA1c) level ≥ 6.5%, (d) a fasting blood glucose level ≥ 7.0 mmol/L, (e) a random blood glucose level ≥ 11.1 mmol/L, or (f) a two-hour blood glucose level ≥ 11.1 mmol/L following an oral glucose tolerance test (OGTT). Depression was defined as PHQ-9 greater than 10, which has been previously validated (Kroenke et al., 2001).

### 2.5 Statistical analysis

Statistical analysis was performed using R software, specifically version 4.3.2. Due to the complex sampling design, all analyses were conducted through weighted data to ensure nationally representative estimates. The current study constructed new sample weights by dividing the original 2-year sample weight by 2. We categorized the participants’ DII into quartiles, with the lowest level designated as the reference group (Q1), and compared the other groups accordingly. The baseline characteristics of participants across the quartiles were compared. Continuous variables were presented as means with standard error (SE) and compared using analysis of variance (ANOVA) for normally distributed variables, otherwise, adopting Kruskal-Wallis test for non-normal distributed variables. Categorical variables were described using frequencies (n) and percentages (%) and compared using Chi-square tests. Cognitive performance based on three tests was analyzed as a binary variable. To examine the association between DII and low cognitive function, a multivariate logistic regression analysis was conducted. Restricted cubic splines (RCS) were employed to flexibly depict the relationship between DII and cognitive performance. If nonlinearity was detected, piecewise regression analysis was applied to examine the inflection point. Subgroup analysis was performed for covariates such as age and gender. All the statistical tests performed as two-sided, and value of *p* <0.05 was considered statistically significant.

## 3 Results

### 3.1 Baseline characteristics

This analysis included 947 participants from the NHANES 2011–2014 dataset. Weighted baseline information of the study population based on the quartiles of the DII are presented in Table 1. Of these participants, 466 (45.08%) were male and 481 (54.92%) were female, and the weighted mean (SE) age was 68.93 (0.26) years. The interquartile ranges of DII scores were − 4.22 to 0.31, 0.31 to 1.82, 1.82 to 3.06, and 3.06 to 4.85, respectively. Race, education, PIR, BMI, the prevalence of diabetes, and hypertension were significantly different across the four groups. In addition, the fourth quartile group has a relatively lower cognitive level.

**Table 1:** Weighted baseline characteristics of participants in DII quartiles.

Characteristics	DII					*p*-Value
	Total	Q1 (−4.22, 0.31)	Q2 (0.31, 1.82)	Q3 (1.82, 3.06)	Q4 (3.06, 4.85)	
	(*n* = 947)	(*n* = 237)	(*n* = 237)	(*n* = 236)	(*n* = 237)	
Age, years	68.93 (0.26)	68.46 (0.46)	69.10 (0.37)	68.33 (0.51)	70.02 (0.54)	0.05
Gender (*n*, %)						**<0.001**
Female	481 (54.92)	92 (43.42)	108 (48.45)	136 (64.12)	145 (66.68)	
Male	466 (45.08)	145 (56.58)	129 (51.55)	100 (35.88)	92 (33.32)	
Race (*n*, %)						**0.01**
Mexican American	95 (3.61)	31 (4.14)	19 (2.69)	19 (2.84)	26 (5.06)	
Non-Hispanic Asian	67 (2.59)	29 (4.00)	13 (1.69)	16 (2.57)	9 (2.00)	
Non-Hispanic Black	179 (7.55)	35 (5.15)	31 (5.10)	57 (8.95)	56 (12.08)	
Non-Hispanic White	503 (81.10)	124 (84.32)	141 (83.14)	119 (80.68)	119 (74.89)	
Other Hispanic	87 (2.99)	17 (1.96)	27 (3.67)	21 (2.67)	22 (3.82)	
Other Race - Including Multi-Racial	16 (2.15)	1 (0.43)	6 (3.70)	4 (2.29)	5 (2.15)	
Education (*n*, %)						**0.01**
Below high school	228 (15.80)	39 (8.91)	58 (14.64)	62 (18.70)	69 (22.50)	
High school graduate	227 (22.16)	52 (18.98)	49 (20.84)	60 (23.62)	66 (26.15)	
Above high school	492 (62.04)	146 (72.11)	130 (64.52)	114 (57.68)	102 (51.35)	
PIR (*n*, %)						**0.004**
<1	145 (8.37)	27 (5.71)	36 (6.53)	36 (7.73)	46 (14.93)	
≥1	802 (91.63)	210 (94.29)	201 (93.47)	200 (92.27)	191 (85.07)	
Smoke (*n*, %)						0.54
No	480 (49.74)	110 (49.34)	115 (45.16)	125 (51.57)	130 (53.96)	
Yes	467 (50.26)	127 (50.66)	122 (54.84)	111 (48.43)	107 (46.04)	
Alcohol use (*n*, %)						0.07
No	155 (14.23)	31 (9.74)	36 (12.79)	40 (15.56)	48 (20.18)	
Yes	792 (85.77)	206 (90.26)	201 (87.21)	196 (84.44)	189 (79.82)	
Diabetes (*n*, %)						**< 0.001**
No	599 (68.89)	171 (82.76)	154 (70.64)	142 (66.50)	132 (51.93)	
Yes	348 (31.11)	66 (17.24)	83 (29.36)	94 (33.50)	105 (48.07)	
Hypertension (*n*, %)						**0.02**
No	280 (33.05)	83 (44.10)	73 (31.37)	73 (32.94)	51 (21.33)	
Yes	667 (66.95)	154 (55.90)	164 (68.63)	163 (67.06)	186 (78.67)	
Stroke (*n*, %)						0.15
No	873 (92.49)	224 (95.94)	217 (90.98)	218 (92.00)	214 (90.66)	
Yes	74 (7.51)	13 (4.06)	20 (9.02)	18 (8.00)	23 (9.34)	
Depression (*n*, %)						0.26
No	857 (91.44)	223 (94.21)	210 (91.09)	217 (91.55)	207 (88.26)	
Yes	90 (8.56)	14 (5.79)	27 (8.91)	19 (8.45)	30 (11.74)	
BMI, kg/m^2^	29.37 (0.38)	27.44 (0.47)	29.54 (0.81)	29.98 (0.59)	30.86 (0.60)	**0.004**
CERAD test score	26.62 (0.38)	27.38 (0.53)	26.43 (0.50)	27.01 (0.50)	25.42 (0.72)	0.09
AFT score	18.26 (0.23)	19.62 (0.52)	18.46 (0.58)	17.75 (0.39)	16.89 (0.48)	**0.01**
DSST score	52.28 (0.66)	56.57 (1.36)	54.17 (0.93)	51.22 (1.31)	45.68 (1.66)	**< 0.001**

DII, dietary inflammation index; PIR, poverty income ratio; BMI: body mass index; CERAD, the Consortium to Establish a Registry for Alzheimer’s Disease; AFT, Animal Fluency Test; DSST, Digit Symbol Substitution Test. Bold value: *p*-value is less than 0.05, indicating statistical significance.

### 3.2 The relationship between DII and cognitive impairment

**Figure 2:**
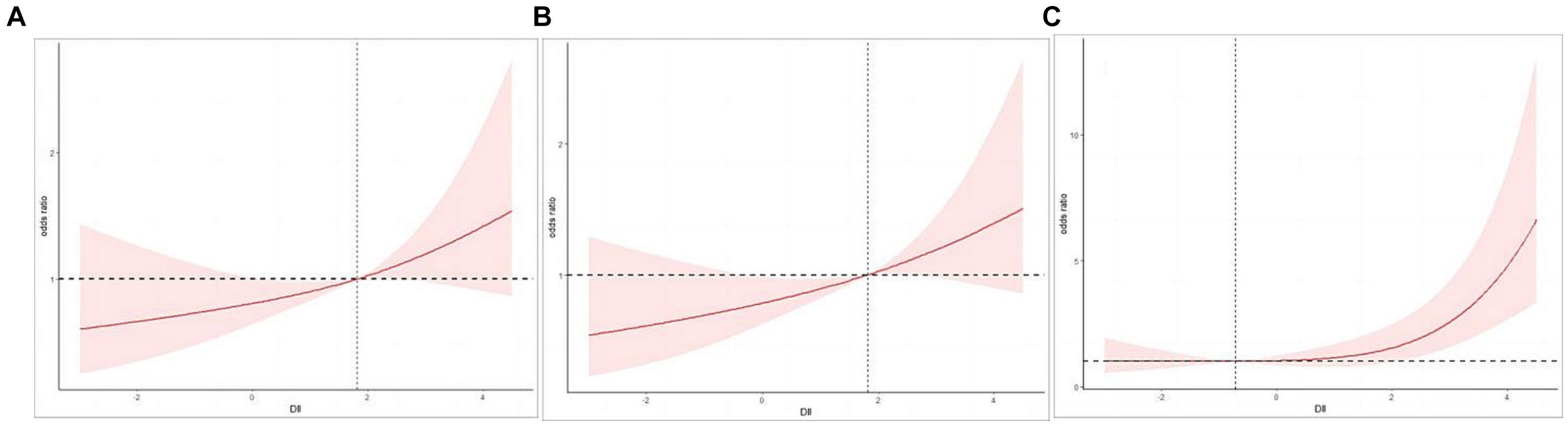
**(A)** Restricted cubic spline (RCS) plot of the association between DII and cognitive impairment evaluated by CERAD. **(B)** Restricted cubic spline (RCS) plot of the association between DII and cognitive impairment evaluated by AFT. **(C)** Restricted cubic spline (RCS) plot of the association between DII and cognitive impairment evaluated by DSST.

Table 2 presents the relationship between DII score and cognitive impairment. Following full adjustment for confounding factors, a strong correlation was observed between DII (continuous) and cognitive decline as assessed by the AFT (OR 1.15, 95% CI 1.02–1.28, *p =* 0.02) and the DSST (OR 1.38, 95% CI 1.13–1.68, *p =* 0.004). However, no significant associations were identified between the continuous DII variable and cognitive decline as assessed by the CERAD test (OR 1.14, 95% CI 0.98–1.32, *p =* 0.09). Transforming DII into quartile variables is aimed at examining the relationships with cognitive impairment across various variable states. For AFT, after adjusting for all covariates (age, gender, race, PIR, BMI, education, smoke, alcohol use, diabetes, hypertension, stroke, and depression), the third and fourth quartile groups showed higher odds than the first quartile (Q3: OR 1.99, 95% CI 1.16–3.41, *p =* 0.02; Q4: OR 1.89, 95% CI 1.05–3.38, *p =* 0.03), which was also confirmed by the trend test (*p* for trend *=* 0.03). For DSST, the fourth quartile had a higher prevalence of cognitive impairment in Crude model (OR 5.16, 95% CI 2.65–10.03, *p* < 0.001), model 1 (OR 5.34, 95% CI 2.50–11.42, *p* < 0.001), and model 2 (OR 4.30, 95% CI 1.85–9.98, *p =* 0.003), when compared to the lowest quartile of DII (*p* for trend = 0.001). Figures 2A,B illustrate the application of smooth curve fitting in order to elucidate the non-linear correlation between DII and cognitive impairment. It showed positive linear relations when adopting CERAD test and AFT. At the same time, a curvilinear association was found between DII and cognitive impairment based on DSST with an inflection point at −0.72 (*p* for nonlinearity = 0.016), as shown in Figure 2C.

**Table 2:** The weighted odds ratio for the relationship between DII and cognitive impairment.

	Crude OR (95% CI)	*p*-Value	Model 1 OR (95% CI)	*p*-Value	Model 2 OR (95% CI)	*p*-Value
CERAD						
Total DII	1.17 (1.05,1.32)	**0.01**	1.19 (1.02,1.39)	**0.02**	1.14 (0.98,1.32)	0.09
Categories						
Q1	Reference		Reference		Reference	
Q2	1.52 (0.90,2.56)	0.11	1.46 (0.84,2.53)	0.17	1.35 (0.76,2.41)	0.27
Q3	1.31 (0.67,2.57)	0.41	1.47 (0.65,3.33)	0.34	1.28 (0.57,2.87)	0.52
Q4	2.21 (1.13,4.33)	**0.02**	2.12 (0.91,4.96)	0.08	1.69 (0.68,4.18)	0.23
*p* for trend		**0.03**		0.08		0.25
AFT						
Total DII	1.22 (1.08,1.37)	**0.002**	1.20 (1.08,1.34)	**0.002**	1.15 (1.02, 1.28)	**0.02**
Categories						
Q1	Reference		Reference		Reference	
Q2	1.75 (0.90,3.40)	0.09	1.82 (0.89,3.72)	0.10	1.68 (0.80,3.55)	0.15
Q3	2.11 (1.28,3.48)	**0.005**	2.25 (1.34,3.77)	**0.004**	1.99 (1.16,3.41)	**0.02**
Q4	2.64 (1.44,4.83)	**0.003**	2.35 (1.31,4.20)	**0.01**	1.89 (1.05,3.38)	**0.03**
*p* for trend		**0.001**		**0.002**		**0.03**
DSST						
Total DII	1.42 (1.21,1.67)	**< 0.001**	1.45 (1.20,1.76)	**< 0.001**	1.38 (1.13, 1.68)	**0.004**
Categories						
Q1	Reference		Reference		Reference	
Q2	1.44 (0.78, 2.66)	0.24	1.43 (0.72, 2.83)	0.29	1.08 (0.48, 2.44)	0.84
Q3	2.14 (0.98, 4.71)	0.06	2.53 (0.96, 6.69)	0.06	2.06 (0.77, 5.56)	0.14
Q4	5.16 (2.65,10.03)	**<0.001**	5.34 (2.50,11.42)	**<0.001**	4.30 (1.85, 9.98)	**0.003**
*p* for trend		**< 0.001**		**< 0.001**		**0.001**

Crude: no covariates were adjusted. Model 1: age, gender, and race were adjusted. Model 2: age, gender, race, PIR, BMI, education, smoke, alcohol use, diabetes, hypertension, stroke, and depression were adjusted. Abbreviations: DII, dietary inflammation index; CERAD, the Consortium to Establish a Registry for Alzheimer’s Disease; AFT, Animal Fluency test; DSST, Digit Symbol Substitution Test; OR, odds ratio; CI, confidence interval. Bold value: *p*-value is less than 0.05, indicating statistical significance.

### 3.3 Subgroup analyses

Table 3 presents the findings from subgroup analyses investigating the link between DII and cognitive impairment in different age and gender cohorts. After adjusting for potential confounders, a positive association in females was observed when conducting AFT (OR 1.15, 95% CI 1.01–1.32, *p =* 0.04) and DSST (OR 1.44, 95% CI 1.15–1.81, *p =* 0.004). Furthermore, when using the DSST as an assessment tool, the current study revealed a higher susceptibility to cognitive impairment among participants over 75 years old (OR 1.40, 95% CI 1.09–1.79, *p* = 0.01). Meanwhile, the results of the interaction test did not reach statistical significance for age and gender (*p* for interaction >0.05), suggesting that the link between DII and cognitive decline remained consistent across different age groups and genders.

**Table 3:** Subgroup analyses for each cognitive test and DII.

	OR (95% CI)	*p*-Value	*p* for interaction
CERAD			
Gender			0.24
Male	1.208 (0.998, 1.462)	0.052	
Female	1.052 (0.859, 1.289)	0.598	
Age, years			0.40
>75	1.11 (0.90,1.37)	0.31	
60–75	1.18 (0.97,1.42)	0.09	
AFT			
Gender			0.95
Male	1.16 (0.99, 1.35)	0.06	
Female	1.15 (1.01, 1.32)	**0.04**	
Age, years			0.17
>75	1.23 (0.97, 1.55)	0.08	
60–75	1.10 (0.96, 1.25)	0.14	
DSST			
Gender			0.67
Male	1.312 (1.068, 1.613)	**0.013**	
Female	1.440 (1.146, 1.810)	**0.004**	
Age, years			0.62
>75	1.40 (1.09, 1.79)	**0.01**	
60–75	1.34 (1.04, 1.71)	**0.02**	

DII, dietary inflammation index; CERAD, the Consortium to Establish a Registry for Alzheimer’s Disease; AFT, Animal Fluency test; DSST, Digit Symbol Substitution Test; OR: odds ratio; CI: confidence interval. Bold value: *p*-value is less than 0.05, indicating statistical significance.

## 4 Discussion

The present study suggests that the risk of cognitive impairment increases with higher DII scores among elderly individuals, and the robustness of the findings was confirmed through adjustment for various potential confounding variables. Furthermore, this affirmative correlation has been substantiated through smooth curve fitting. DII scores are non-linearly associated with attention, processing speed, and working memory capacity. The relationship can be described as follows: The risk of cognitive impairment increases significantly with the increment of the pro-inflammatory effect of the diet when DII exceeds −0.72. Additionally, individuals aged over 75 years, or females are more prone to suffer from cognitive impairment with a high inflammatory diet.

To date, there have been limited endeavors to explore the correlation between DII scores and cognitive performance in extensive, representative populations. Hayden et al. conducted a prospective cohort study based on 7,085 participants aged 65–79 years to find that higher DII values were associated with greater cognitive decline and an increased risk of incident mild cognitive impairment (MCI) (Hayden et al., 2017). Another three cross-sectional studies reached similar conclusions (Frith et al., 2018; Shin et al., 2018; Sun et al., 2022). Consistent with previous studies, our data demonstrate that a pro-inflammatory diet is related to low cognitive performance. In addition to this, this study found a dose–response relationship when evaluating the association of executive function. When participants were stratified by gender and age in this study, a notable negative correlation was observed between DII score and cognitive function was found in females and persons aged above 75.

The underlying mechanisms that connect dietary patterns to cognitive function are still ambiguous, but inflammatory pathways and gut microbiome composition are likely to be involved. Currently, there’s a broad consensus that a rise in inflammatory indicators in the body correlates with neural decay, and this is thought to exacerbate inflammation in neural tissue through neural and endocrine routes (Koyama et al., 2013). Featuring a higher level of inflammatory markers such as interleukin-6 (IL-6), tumor necrosis factor-α (TNF-α), and C-reactive protein (CRP), a pro-inflammatory diet is assumed to pose a certain extent impact of systemic inflammation on cognitive performance, which may interact with the central nervous system (CNS) in blood brain barrier (BBB)-dependent and independent pathways (Fung et al., 2012). Specifically, cytokines are capable of traversing the BBB through particular transport proteins or receptors. Concurrently, prostaglandins that are synthesized in reaction to cytokine activity contribute to the enhancement of the BBB permeability as well as the transit of leucocytes through the BBB. Besides, cytokines have the potential to stimulate afferent nerves, such as the vagus nerve, which in turn convey inflammatory signals to the brainstem (McCusker and Kelley, 2013). The gut microbiome also plays a key role in intestinal permeability and immune regulation. Approximately 60% of the variations within the gut microbiome can be ascribed to dietary factors (Jin et al., 2019). Dysbiosis prompts the release of endotoxins, such as lipopolysaccharides (LPSs) and microbial amyloids which can weaken the intestinal barrier and boost the presence of inflammatory cytokines in the bloodstream, furthermore, disrupting the integrity of the BBB and initiating alterations in neuroinflammation by activating microglia and obstructing amyloid clearance (Pistollato et al., 2018). The aforementioned mechanisms provide the premise that inflammation may mediate the relationship between pro-inflammatory diet and cognitive changes.

As is well known, cognition involves the collaborative efforts of multiple brain regions, with the prefrontal and parietal lobes serving as the primary regulators of cognitive functions. These areas play a crucial role in learning, memory, decision-making, planning, problem-solving, and executive functions. Diet-induced inflammation can impact multiple areas of the brain. Previous animal research reported that a high-fat diet (pro-inflammatory) can lead to microglial activation and neuroinflammation in the prefrontal cortex and hippocampus (Yang et al., 2020; Sood et al., 2023). During a randomized, cross-over study, Sartorius et al. discovered through resting-state functional MRI that increased consumption of saturated fatty acids, known for their pro-inflammatory effects, led to lower resting activity in both the hippocampus and the inferior parietal cortex, whereas a comparable intake of monounsaturated fatty acids, known for their anti-inflammatory effect, acted as a positive modulator of brain activity in humans (Sartorius et al., 2012). Based on the results of the present study, it was found that after adjusting for included covariates, there is a positive correlation between DII scores and cognitive impairment reflected by AFT and DSST. The AFT assesses language production and logical thinking abilities, while the DSST evaluates attention, processing speed, and working memory capacity. Therefore, this study infers that the inflammatory effects of a pro-inflammatory diet may extend to brain regions including the prefrontal lobe, parietal lobe, hippocampus, and Broca’s area, which is responsible for language expression. However, prospective dietary intervention trials are needed to further validate the above inference.

A strong positive association of DII with low cognitive function was detected among females and elderly people over 75 years. As the women enrolled in this study were over 60 years old, the majority of them were experiencing the menopausal period. Many researches have indicated a connection between estrogen and impairments in cognitive abilities (Schwarz et al., 2023; Sochocka et al., 2023). Primarily, the mechanism for this cognitive impairment is believed to stem from the direct impact of diminished estrogen concentrations on neural cells. Research on female rodent models indicates that reduced estrogen is associated with synaptic loss, lower neural connectivity, and neuron damage (Chen et al., 2023; Meng et al., 2023). Concurrently, another pathway that might contribute to cognitive decline is cerebral inflammation. It has been observed that when the production of estrogen lessens during menopause, there is a marked rise in inflammatory indicators such as IL-6, IL-1, and TNF-α. In addition, it has been shown that estrogen can dampen the reaction of microglia and pro-inflammatory cytokines to LPS, achieved through the hindrance of the NF-κB signaling cascade (Pozzi et al., 2006; Gatson et al., 2009). Microglial macrophages in the brain become chronically activated during aging (Dilger and Johnson, 2008), promoting sustained production of pro-inflammatory cytokines (Heppner et al., 2015; Leonardo and Fregni, 2023). These molecules can trigger a cascade of detrimental effects on the brain, leading to amyloidosis, neuron loss, brain shrinkage, and assorted issues linked to vascular health in the brain, like minor hemorrhages, strokes, and neurodegeneration (Gu et al., 2017; Swardfager et al., 2017; Gu et al., 2019).

This study has identified a positive correlation between DII and cognitive impairment, suggesting that beyond potential pathophysiological mechanisms, social economic and psychological factors should also be taken into account. The data indicate that as DII levels increase, the number of individuals with PIR less than 1 also increases, with a significant statistical difference observed. Foods with a high DII tend to be cheaper and thus more accessible to those with lower incomes. People with cognitive impairments, whose condition may have affected their ability to work, maybe more inclined to purchase lower-priced foods, which often contain high levels of processed products, saturated fats, and sugars. Cognitive impairment might also inherently impact an individual’s capability to make healthy dietary choices. For instance, difficulties with decision-making or reduced problem-solving skills could interfere with the ability to plan and prepare healthy meals. Additionally, diets high in DII are generally more palatable, including foods that are rich in sugars and fats. Individuals with cognitive impairments might be less influenced by health information, making them more likely to choose foods based on taste preferences rather than considerations of health.

This study offers multiple strengths. Initially, the study utilized a substantial sample of elderly participants from across the U.S., which enhanced the statistical power to provide a robust result. Second, accounting for various possible potential confounding factors while examining the links between DII and cognitive decline, the study eliminated as many bias-inducing factors as possible to ensure more reliable results. Third, the study investigated the non-linear relationship with a dose–response relationship suggesting food inflammatory potential beyond the threshold would contribute to the pathogenesis of cognition impairment.

The study has certain constraints. Firstly, the cross-sectional nature means causality between DII and cognitive impairment cannot be established. In the future, prospective longitudinal analyses will be vital to validate our findings. Secondly, despite comprehensive adjustments for confounders, the exclusion of all external variables is not absolute. Thirdly, evaluating cognitive abilities is a complex process and it is optimal to employ a variety of approaches for a thorough assessment. In this study, the cognitive tests, due to the limitations of the NHANES database, did not cover all domains of cognition. Furthermore, the reliance on 24-h dietary recall may miss chronic dietary habits and introduce recall bias, potentially skewing the true DII scores. Nonetheless, the research provides a comprehensive examination of the association between the inflammatory potential of diet and cognitive function among the United States elders.

## 5 Conclusion

These analyses uncovered a positive correlation between a diet with high inflammatory potential and low cognitive performance, particularly in attention, processing speed, and working memory capacity. Attention to diets that promote inflammation is particularly crucial for women and older adults over 75. Dietary modifications may have the potential to improve cognitive function in the elderly population. However, additional research into nutritional interventions is necessary to explore changes in the nervous system linked to diet.

## Data availability statement

The datasets presented in this study can be found in online repositories: https://www.cdc.gov/nchs/nhanes/.

## Ethics statement

The NHANES obtained approval from the Ethics Review Board of the American National Center for Health Statistics. The studies were conducted in accordance with the local legislation and institutional requirements. The participants provided their written informed consent to participate in this study.

## Author contributions

YZ: Data curation, Formal analysis, Software, Writing – original draft. YP: Data curation, Formal analysis, Software, Writing – original draft. WD: Conceptualization, Formal Analysis, Writing – original draft. QX: Data curation, Software, Writing – original draft. WZ: Conceptualization, Methodology, Writing – review & editing. ML: Conceptualization, Methodology, Writing – review & editing.
